# *C1ql1* is expressed in adult outer hair cells of the cochlea in a tonotopic gradient

**DOI:** 10.1371/journal.pone.0251412

**Published:** 2021-05-12

**Authors:** Joyshree Biswas, Robert S. Pijewski, Rohit Makol, Tania G. Miramontes, Brianna L. Thompson, Lyndsay C. Kresic, Alice L. Burghard, Douglas L. Oliver, David C. Martinelli

**Affiliations:** 1 Department of Neuroscience, University of Connecticut Health, Farmington, CT, United States of America; 2 The Connecticut Institute for the Brain and Cognitive Sciences (IBACS), Storrs, CT, United States of America; University of Michigan, UNITED STATES

## Abstract

Hearing depends on the transduction of sounds into neural signals by the inner hair cells of the cochlea. Cochleae also have outer hair cells with unique electromotile properties that increase auditory sensitivity, but they are particularly susceptible to damage by intense noise exposure, ototoxic drugs, and aging. Although the outer hair cells have synapses on afferent neurons that project to the brain, the function of this neuronal circuit is unclear. Here, we created a novel mouse allele that inserts a fluorescent reporter at the *C1ql1* locus which revealed gene expression in the outer hair cells and allowed creation of outer hair cell-specific *C1ql1* knockout mice. We found that *C1ql1* expression in outer hair cells corresponds to areas with the most sensitive frequencies of the mouse audiogram, and that it has an unexpected adolescence-onset developmental timing. No expression was observed in the inner hair cells. Since C1QL1 in the brain is made by neurons, transported anterogradely in axons, and functions in the synaptic cleft, C1QL1 may serve a similar function at the outer hair cell afferent synapse. Histological analyses revealed that *C1ql1* conditional knockout cochleae may have reduced outer hair cell afferent synapse maintenance. However, auditory behavioral and physiological assays did not reveal a compelling phenotype. Nonetheless, this study identifies a potentially useful gene expressed in the cochlea and opens the door for future studies aimed at elucidating the function of C1QL1 and the function of the outer hair cell and its afferent neurons.

## Introduction

The organ of Corti, located in the cochlea, houses two types of sensory hair cells: inner hair cells (IHCs) and outer hair cells (OHCs). Both have efferent and afferent neuronal connections to facilitate communication with the brain. IHCs are responsible for the basic aspects of acoustic sensory transduction and connect to the brain by type I spiral ganglion neurons (SGNs) [1]. A single type I SGN dendrite receives synaptic input from a single IHC, and ~95% of all SGNs are type I; thus, they are responsible for the large majority of auditory input [2, 3]. In contrast, OHCs are known for their mammalian-specific adaptation of ‘electromotility’, in which sound-evoked depolarization causes the OHC body to change its length/stiffness, producing force [4–6]. The result is a relative enhancement of frequency selectivity and an effect commonly described as ‘cochlear amplification,’ allowing increased auditory sensitivity of low-level sounds [7, 8].

OHCs have an afferent connection to type II SGNs, but the function of this afferent activation remains mysterious [9, 10]. In contrast to IHCs, multiple OHCs innervate a single type II SGN [11–17]. OHCs have a smaller number of synaptic vesicles per synapse, smaller vesicle release probability, and smaller numbers of ribbon synapses per hair cell; moreover, axons of type II SGNs make up only about 5% of the auditory nerve [17–24]. Thus, OHC afferent activity must play a far different role in auditory sensory transduction than IHC afferents. The OHC afferents could potentially function in the detection of and/or protection against high intensity sounds with potential to cause OHC damage [25, 26].

This present study attempts to describe the expression and function of the potential synaptic adhesion protein C1QL1 in the auditory system, and was prompted by a gene expression microarray study revealing that *C1ql1* is expressed specifically in OHCs, and not IHCs [27]. This conclusion was reproduced with single-cell RNA sequencing [28], but interestingly other gene expression studies of cochlear cells did not find *C1ql1* expression in either hair cell type [29, 30]. There are four members of the complement component 1, q subcomponent-like (C1QL1-4) protein family, which are expressed in small subpopulations of CNS neurons [31–34]. C1QLs likely have no function in the complement system, and should not be confused with another member of the superfamily, C1q [35].

Published work on the C1QL family paints a rough consensus of their function [31, 32, 36–39]. C1QLs are transported down axons and secreted pre-synaptically, where they bind to a post-synaptically localized G protein-coupled receptor (GPCR) called adhesion G protein-coupled receptor B3 (ADGRB3; a.k.a. BAI3). The likely consequence of this interaction is to promote excitatory synapse formation and/or maintenance, possibly by completing a trans-synaptic adhesion complex or possibly by activating ADGRB3’s GPCR signaling [34]. Note that C1QLs likely have additional functions as well: 1) C1QL2 and C1QL3 are expressed in the dentate gyrus granule neurons but do not have a function in synapse density there [40], 2) C1QL3 is expressed in neural stem cells of the sub-ependymal zone with an unknown function [41], and 3) C1QL3 (a.k.a. CTRP13) is expressed in adipose tissue and likely engages in paracrine signaling [42, 43]. Regardless, *C1ql1* and *C1ql3* knockout mice have reduced synapse density in each brain region studied where ADGRB3 and a C1QL are both highly expressed. C1QL1 is particularly fascinating: in addition to its 98% amino acid conservation between human and mouse orthologs, its mRNA belongs to a very rare group of transcripts in which post-transcriptional RNA editing is conserved between human and mouse [44, 45], suggestive of deeply conserved function.

Our investigation into *C1ql1* expression in the cochlea revealed expression in OHCs that has both an intriguing tonotopic gradient pattern, and an unexpected adolescence-onset developmental timing. Based on C1QL1 secretion in the brain, we predict that C1QL1 will be secreted into the synaptic cleft where it may have a function in the formation, maintenance, or physiology of the synapse connecting the OHC with the type II SGN. To study the function of C1QL1 in the cochlea, we created a novel *C1ql1* conditional knockout allele and OHC-specific *C1ql1* knockout mice. Various assays in auditory physiology, behavior, and histology did not reveal any reproducible and statistically significant phenotypes in these conditional KO mice, so the function of C1QL1 in the cochlea remains unclear. Nonetheless, this study identifies a potentially useful gene expressed in the cochlea and opens the door to future studies aimed at elucidating the function of C1QL1 and possibly the function of the mysterious type II SGNs.

## Materials and methods

This study was carried out in strict accordance with the recommendations in the Guide for the Care and Use of Laboratory Animals of the National Institutes of Health. The protocol was approved by the UConn Institutional Animal Care and Use Committee (IACUC) (Protocol Number: 102047–0322). All surgery was performed under ketamine/xylazine anesthesia, and all efforts were made to minimize suffering.

### *C1ql1* conditional knockout allele with mVenus reporter

**Fig 1:**
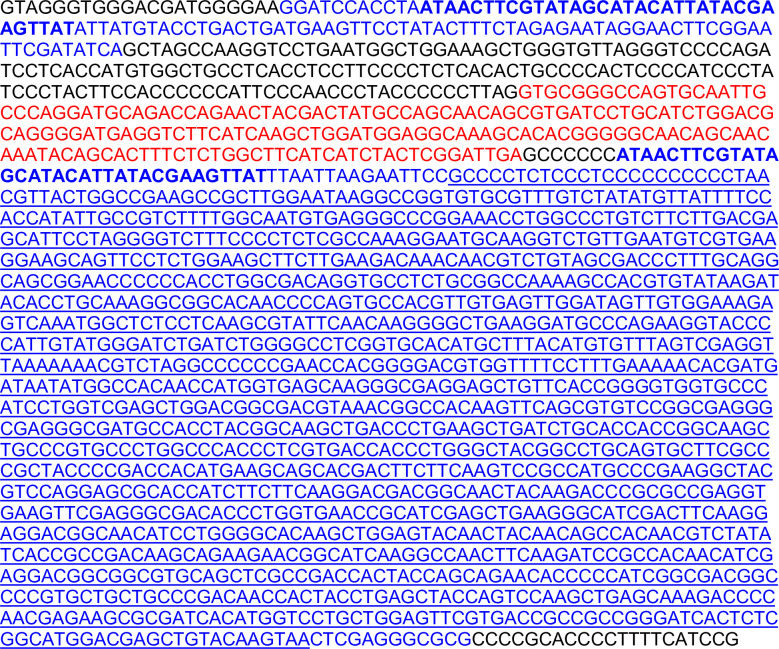
Sequence of *C1ql1* conditional mutant allele. Sequence of the novel *C1ql1* allele, (after the neomycin resistance cassette was removed) beginning partway through the intron. In black is the original non-coding *C1ql1* allele, in red is the open reading frame (ORF) for exon 2, in blue are bases added to the allele with loxP sites bolded and IRES-mVenus underlined.

This allele was created simultaneously with the previously published *C1ql3* conditional knockout allele with mVenus reporter, using an identical strategy [31]. The targeting vector was constructed using the recombineering (recombination-mediated genetic engineering) technique as described in [46]. A 9,630 bp genomic DNA fragment containing exon 2 of the *C1ql1* gene was retrieved from BAC clone RP23-227G4. A frt-PGKNeo-frt cassette was inserted 150 bp upstream of exon 2, and a loxP site was inserted after the stop codon. Thus, a fragment of 339 bp genomic DNA containing exon 2 coding region was floxed followed by an IRES mVenus and endogenous 3’ UTR. The homologous arms of the construct were 5,432 bp and 3,860 bp, respectively. The targeting vector was electroporated into G1 embryonic stem (ES) cells which were derived from F1 hybrid blastocysts of 129S6 x C57BL/6J crosses. G418-resistant ES colonies were isolated and screened by nested PCR using primers outside the construct paired with primers inside the construct. The sequences for primers used for ES cell screening were as follows: 5’ arm forward primers: *C1ql1* scr F1 (5’-tagtcagatcacagggctct-3’) and *C1ql1* scr F2 (5’-gctaagattcaagctgacgc-3’). Reverse primers: Mut scr 5R1 (5’-TTCTGAGGCGGAAAGAACCA-3’) and loxp scr 5R2 (5’-GGAACTTCATCAGTCAGGTA-3’). 3’ arm forward primers: mVenus scr 3F1 (5’-tgagctaccagtccaagctg-3’) and mVenus scr F2, (5’-catggacgagctgtacaagt-3’). Reverse primers: *C1ql1* scr 3R1 (5’-GAATCCACCCATTCTGGGAT-3’) and *C1ql1* scr 3R2 (5’-ACAACTGGGTGGCCTATGGA-3’). The ES clones with both arms positive were identified for generation of chimeric mice. Chimeric mice were generated by aggregating ES cells with 8-cell embryos of CD-1 strain. The correct targeting was further confirmed by a homozygosity test in progenies. The neomycin resistance cassette was removed by breeding to mice expressing FLP recombinase [47] and the Flp transgene was bred out before experiments began. The sequence of this allele shown in Fig 1. Mice harboring the novel allele were back-crossed to the C57BL6/J strain. For genotyping, 3 primers were used in 1 reaction: *C1ql1*-F 5’- CAGTGCAATTGCCCAGGATG, *C1ql1*-R 5’- GGCTAAGGACAATTCAGCCT, and mVenus-R 5’- TGAGCTACCAGTCCAAGCTG. The PCR program protocol was 94°C for 3 min, 94°C for 15 sec, 55°C for 15 sec, 72°C for 18 sec, and steps 2–4 were repeated 34 times. The mutant band was 314 bp whereas the WT band was 262 bp long.

### Creation of *C1ql1* conditional knockouts

To create *C1ql1* conditional knockouts, mice harboring the *C1ql1*^*flox*^ allele were bred to mice expressing Cre recombinase under control of the endogenous *Gfi1* promoter (*Gfi1*^*Cre*^) [48]. Similar to the original publication, we found that mice homozygous for the *Gfi1*^*Cre*^ allele have reduced viability and are deaf, and thus could not be used for experiments or breeding. To produce mice for experiments, double heterozygotes were bred to create control (*C1ql1*^*+/+*^;*Gfi1*^*+/Cre*^) and experimental or conditional knockout (*C1ql1*^*flox/flox*^;*Gfi1*^*+/Cre*^) mice. For *Gfi1*^*Cre*^ genotyping, 4 primers were added in 1 reaction: *Gfi1*-F 5’- GGGATAACGGACCAGTTG, *Gfi1*-R 5’- CCGAGGGGCGTTAGGATA, *Cre*-F 5’- GACCAGGTTCGTTCACTCATG, and *Cre*-R 5’- AGACCAGGCCAGGTATCTCT. Mutant band = 435 bp, WT band = 610 bp. Denville Choice Taq Mastermix (CB4070-8) was used. PCR program: 94°C for 3 min, 94°C for 15 sec, 56°C for 15 sec, 72°C for 37 sec, repeat to step 2 34 times.

### Histology and immunofluorescence

We adapted the histology protocol from [10]. Unfixed cochleae were removed from mice, perfused with 1 ml of 4% paraformaldehyde (PFA) through the round and oval windows, then fixed for an additional 2 hours in PFA with gentle agitation. After washes with 1X phosphate buffered saline (PBS, pH 7.4) (3x 10 min each), cochleae were decalcified for 2 days in 5 ml of EDTA (171 mM in water). After washing with PBS (3x 10 min each), cochleae were switched to a solution of 30% sucrose in PBS (2 washes with sucrose solution, 15 min each) and frozen on dry ice and stored at -80°C until needed. Once thawed and washed with PBS (3x 10 min each), pieces of organ of Corti were dissected in a solution of PBS with 0.1% Triton X-100 detergent (detergent was needed to prevent dissected pieces from sticking to plastic). Whole mount immunofluorescence of organ of Corti pieces began in a blocking solution of 5% goat serum and 0.3% Triton X-100 in PBS for 60 min at room temperature. An upside-down cap from a 1.5 ml tube worked as a convenient well in which to place organ of Corti pieces. Primary antibodies were diluted in blocking solution and applied overnight at room temperature with gentle agitation. Pieces were washed with 1X PBS + 0.3% Triton X-100 (3x 5 min each) followed by application of appropriate secondary antibodies diluted in blocking buffer for 1 hour at room temperature with gentle agitation. After 3 more washes in 1X PBS, pieces were mounted on glass slides with DAPI Fluoromount-G (SouthernBiotech).

The following antibodies were used: mouse anti-CtBP2 (1:200, BD Biosciences #612044), guinea pig anti-VAChT (1:500, Synaptic Systems #139 105), chicken anti-parvalbumin (1:500, Synaptic Systems #195 006), and rabbit anti-BAI3 (a.k.a. ADGRB3, 1:100, A323 created in [49]). Although not depicted in this manuscript, we also report that mouse anti-parvalbumin (Millipore MAB1572) works equally well in the cochlea. The signal intensity of mVenus from the *C1ql1*^*flox*^ allele was low so an anti-GFP antibody was used to enhance signal; rabbit anti-GFP (1:1000, A11122 Invitrogen). Secondary Alexa Fluor antibodies (1:400, Life Technologies; all made in goat) were used with appropriate fluorescent conjugates.

Low resolution fluorescent images were taken with a 10x objective using a BZ-X810 Keyence All-in-one fluorescence microscope. High magnification image z-stacks were taken with a 63x objective using a Zeiss LSM 880 confocal microscope. For the quantification of synaptic puncta: puncta were counted by hand in the Zeiss ZEN 2.3 software by an independent observer blinded to genotypes.

To quantify the mVenus fluorescence intensity of representative frequency regions, pictures were cropped to be 13 hair cells wide, and the mean pixel intensity of each hair cell was measured using ImageJ software. Mean fluorescence values were converted to a percentage for each mouse, where the lowest OHC fluorescence for that mouse was 0% and the highest value for that same mouse was 100%. Any OHC with a value greater than 66% was characterized as “high” mVenus expression, between 33% and 66% was “medium”, between 33% and 15% was “low”, and less than 15% was “none”.

### Distortion product otoacoustic emission

Otoacoustic emissions were recorded according to the recommendations provided by Tucker-Davis Technologies, Inc. for the System 3 RZ6 and BioSigRZ software. Mice were anesthetized using a ketamine/xylazine (10 mg/ml / 1.42 mg/ml) cocktail. A rectal temperature monitoring system was used to maintain body temperature at 37°C. Sounds were delivered into the ears in a closed field setup using magnetic speakers and an ER-10B+ microphone probe. Only one ear was assayed for each mouse—whether it was the left or right ear was randomized. A plastic 200 μL pipette tip was cut and one end was placed on the probe and the other placed into the mouse’s ear canal as it lays on its side with the magnetic speakers suspended over the mouse’s head. Silicon tubes of length 5 cm were used to connect the MF-1 speakers to the probe. The center frequencies tested were 4, 8, 12, 16, 24, and 32 kHz calibrated from 2–40 kHz for each ear per mouse. The F2/F1 frequency ratio was 1.2; for example, for center frequency 4 kHz, F1 = 3.636 kHz and F2 = 4.36 kHz. The stimuli were two tones L1 and L2 (the corresponding intensity levels to the frequencies F1 and F2) presented continuously. L1 and L2 were at a 10 dB difference, L1 beginning at 90 dB sound pressure level (SPL; thus L2 at 80 dB SPL) decreasing to 20 dB SPL in 5 dB steps. Emission peaks were measured at 2F1 –F2. Emission intensities for each group were averaged and plotted as emission dB SPL vs. L2 stimulus level.

### Auditory brainstem response for thresholds

Immediately after otoacoustic emissions were measured, click auditory brainstem responses were recorded to determine overall threshold. Three electrodes were inserted under the skin, the active electrode along the midline of the animal’s scalp, the ground electrode under the animal’s left ear, and the reference electrode under the right ear. The same BioSigRZ software was used for measurements as well as the RZ6 in combination with a TDT RA4PA medusa preamp with TDT RA4LI low impedance headstage. Sound stimuli were delivered open field using an electrostatic speaker (Revelator R2904/7000-05 Tweeter, ScanSpeak, Denmark) placed 9 cm above and along the midline of the animal’s head. Stimuli were 0.2 millisecond clicks presented at 21 Hz. Stimulus level increased from 0 to 90 dB SPL in increments of 5 dB until the threshold was reached. A 20 dB SPL gain was added. The responses were band-pass filtered between 500–3000 Hz and the lowest intensity where the 5 expected identifiable wave forms were identified was the criterion to determine the threshold. The genotype of the mouse was always blinded to the experimenter.

### Amplitude-modulated frequency response

Immediately after click auditory brainstem responses were measured, audiograms were measured for each mouse. The same open field speaker arrangement and electrode placement were used as described above. The general amplitude-modulated frequency response principle is described in [50, 51] and performed herein as described in [52]. In brief, the detection is based on the magnitude squared coherence (MSC) algorithm calculated with modulation sounds and the recorded responses of the electrical potential produced by the brain. The statistical detection algorithm was implemented with the use of a MATLAB-based software program running on a PC, and data were recorded via the same TDT system described above. Carrier frequencies tested were 2, 4, 8, 12, 16, 24, 32, and 40 kHz with stimulus levels starting at 90 dB SPL decreasing in 5 dB steps. The modulation frequency was 42.9 Hz, with 100% modulation depth using a cosine exponent 8 envelope. The criteria for a successful response of a sound stimulus was defined as either 1) a MSC value greater than 0.25 and a MSC strength greater than 3; or 2) a MSC value greater than 0.5. A ‘passed’ detection of the sound stimulus was obtained after 5 consecutive blocks of successful responses (1 block had 8 epochs, and 1 epoch had 10 modulation cycles or 250 ms, whichever was longer). A ‘failed’ detection of the sound stimulus was defined as the above criteria not being met in 5 consecutive blocks after 350 epochs. The threshold was determined at the intensity level before two consecutive failed detections.

### Acoustic trauma

A pilot experiment was conducted using 8-week-old *C1ql1*^*flox/flox*^ mice to establish an acoustic trauma protocol. Mice were anesthetized with ketamine/xylazine and placed in a sound-proof chamber (Industrial Acoustics Company, Inc). The dimensions of the chamber (W x H x D) were 78 x 61 x 56 cm. In the chamber, a JBL Professional Series speaker, model # 2446H with impedance of 8 ohms, was mounted with the bell of the speaker 10 cm away from the subject. The sound presented was an octave-wide stimulus with frequencies between 8–16 kHz. Mice were subjected to the noxious noise for varying durations, ranging from 0–60 minutes (n = 3 for each duration). Maximum trauma was achieved at 20 min and longer durations are not shown. The intensity of the sound was measured at 120 dB SPL at the tip of the mouse’s snout. Based on the results of this pilot experiment, we selected 2.5 minutes of acoustic trauma to use for experiments on control and cKO mice (see Results section for more details).

To assist with frequency mapping of the dissected cochleae for the counting of surviving OHCs, bright-field images were processed in ImageJ using a frequency mapping plugin from Massachusetts Eye and Ear called ImageJ Plugin for Cochlear Frequency Mapping in Whole Mounts.

### Acoustic startle response

Mice between 7–10 weeks in age were tested for acoustic startle response using an SR Chamber (San Diego Instruments). The series of stimuli were randomized across sixty trials after a five minute acclimation period. During all trials, a fan was on in the chamber to produce a consistent background noise at 70 dB SPL. The intensities of the 50 ms broadband stimuli were: 0, 75, 85, 95, 105, and 115 dB SPL, and ten trials were performed for each stimulus level. Between each sound, there was a random time interval of silence that ranged between five and thirty seconds. Mice were weighed immediately after the startle experiment. A ratio was calculated by computing the averaged startle responses (millivolts, mV) over each of the ten trials, and dividing by the weight of the subject.

### Pre-pulse inhibition of the acoustic startle response

Mice 10 weeks in age were tested using an SR Chamber (San Diego Instruments). A series of 70 acoustic stimuli were presented after a five minute acclimation period. Between each test stimulus, there was a random time interval of silence that ranged between five and thirty seconds. During all trials, a fan was on in the chamber to produce a consistent background noise at 70 dB SPL. Trials 1–10 and 61–70 were 115 dB SPL for 50 ms at broadband frequencies. The average of each 10-trial block was used to calculate percent habituation to the startle response. Trials 11–60 were used to measure the effect of pre-pulse inhibition to the acoustic startle response. Each startle stimulus was always 115 dB SPL, with a 20 ms broadband pre-pulse presented at 0, 71, 75, 81, or 85 dB SPL in a random order, with 10 total trials of each. The 10 trials of each were averaged. There were 10 ms silence between the pre-stimulus and test stimulus. The percent pre-pulse inhibition was calculated using the following formula (for example at 71 dB SPL pre-pulse): 100-(100*y/x); where x = startle with 0 dB pre-pulse, y = startle with 71 dB SPL pre-pulse).

### Active sound avoidance paradigm

A custom sound avoidance behavior apparatus was built based on the general design described for rats in [53]. The outside dimensions of the sound-attenuated outer chamber (W x H x D) were 60 x 60 x 61 cm. Dimensions inside the dark chamber after sound-proof padding glued to inner walls (W x H x D): 48 x 48 x 49.5 cm. An inner chamber to contain the mouse was created from metal fencing (19 x 19 x 19 cm) which was adjacent to a JBL Harman C-6IC 6.5” In-Ceiling & In-Wall Loudspeaker. If a mouse was inside this inner chamber, the maximum possible distance between its ears and the speaker was 19 cm. The inside of the chamber had an ambient sound intensity level of 53 dB SPL measured with a Digital Sound Level Meter (RadioShack 33–2055) set to C-weighting. When the speaker was switched on, it would emit 3 possible sound intensity levels (8–16 kHz): 86, 93.5, and 101 dB SPL (average sound intensity level inside the inner chamber). An ‘escape’ tunnel was created allowing the mouse to leave the inner chamber if desired, although this would bring the mouse out of the dark inner chamber and into bright light (1000–2000 lux). A mounted camera recorded the movement of the mouse if it left the inner chamber.

Mice were first habituated in the chamber for 3 sessions over 3 days. Habituation days consisted of 10 minutes in the apparatus in a blacked-out room with lights off, followed by 10 minutes with the lights on. Any mouse that explored outside the inner darkened chamber for less than 5% of the sessions with the lights on was excluded for further analysis. Following the habituation sessions, mice were given 3 testing sessions, also on 3 consecutive days. Test days consisted of 10 minutes with no sound and 10 minutes each at 3 different decibel levels for a total of 40 minutes in the apparatus. The order of sound stimulus was randomized. The first day the order of decibel level presentations was: off, 101, 86, 93.5 dB SPL. The second day was 86, off, 93.5, 101 dB SPL. The third day was 101, 93.5, off, 86 dB SPL. Idtracker software [54] was used to analyze videos for each decibel level and used to determine the amount of time that was spent in the light portion away from the speaker and the amount of time spent in the dark portion of the apparatus next to the speaker. The 3 values for each of the 3 days at each sound intensity level were averaged to create the final graph.

### Statistical analysis

GraphPad Prism software was used to perform 2-tailed Student’s *t*-tests and repeated measures 2-way ANOVA with Bonferroni’s post-hoc tests. The *p*-value and sample sizes are reported in each figure. Threshold of *p* ≤ 0.05 indicated statistical significance. No statistical significance was observed with any test unless specifically mentioned.

## Results

### *C1ql1* is expressed in a subset of adult OHCs

**Fig 2:**
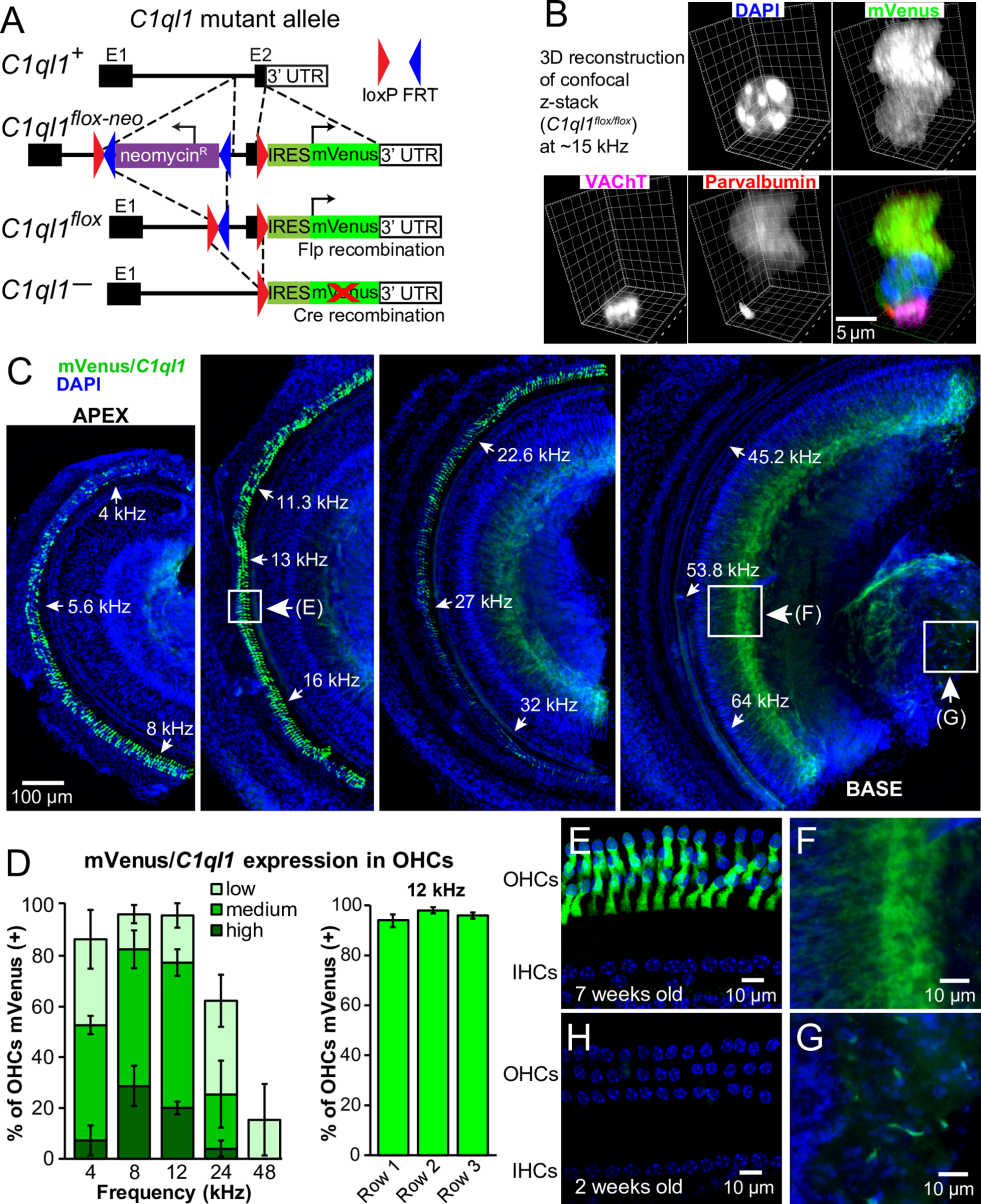
*C1ql1* is expressed by OHCs. (A) Targeting scheme for conditional *C1ql1*-mVenus mutant allele. E1 = exon 1; E2 = exon 2. The red X indicates that after Cre-mediated recombination, mVenus protein is not made due to degradation of the mRNA. All experiments in this manuscript use the version of the allele after Flp-mediated recombination. (B) 3D confocal fluorescence image of a single mVenus-positive OHC from the ~15 kHz region of the organ of Corti from a *C1ql1*^*flox/flox*^ mouse. Immunofluorescence for parvalbumin marks the post-synaptic side of the afferent synapse, VAChT marks the pre-synaptic side of the efferent synapse, and DAPI marks the nucleus. The signal intensity of mVenus from the *C1ql1*^*flox*^ allele was low so an anti-GFP antibody was used to enhance the signal. (C) Representative entire organ of Corti from a 7-week-old *C1ql1*^*flox/flox*^ mouse imaged with a widefield fluorescence microscope. (D) Quantification of frequency regions of OHCs that express *C1ql1*/mVenus. Left: OHCs at 5 representative frequency locations were measured for mVenus fluorescence intensity and categorized as having high, medium, low, or zero signal. N = 5 mice; error bars are standard deviation. Right: the presence of any mVenus was quantified in each of the 3 OHC rows. The representative frequency region of 12 kHz is shown. No differences were noted at any location along the tonotopic gradient. (E) Magnified view of a boxed region in panel C. (F) Magnified view of a boxed region in panel C. (G) Magnified view of a boxed region in panel C. (H) Representative image from a 2-week-old *C1ql1*^*flox/flox*^ mouse taken at similar frequency location as panel E.

To investigate the expression and function of *C1ql1*, we created a novel *C1ql1* allele, in which an essential exon is floxed and an internal ribosome entry site (IRES)-mVenus is knocked into the 3’ UTR (referred to as ‘*C1ql1*^*flox*^’; Fig 2A). Any cell expressing *C1ql1* also simultaneously expresses mVenus. It is important to note that it is not a fusion protein, as the IRES promotes translation of the mVenus separately. This allele also has flanking loxP sites around an essential exon, allowing for conditional knockout potential of C1QL1. Mice homozygous for the conditional *C1ql1* allele (*C1ql1*^*flox/flox*^) were viable, fertile, and showed no observable phenotype.

To test whether *C1ql1* is expressed in OHCs, we dissected organs of Corti from 7-week-old *C1ql1*^*flox/flox*^ mice and examined them for expression of mVenus. We found clear expression of cytoplasmic mVenus in OHCs (Fig 2B), but only in a subset of OHCs (Fig 2C–2E). No expression was ever observed in IHCs. Examining the entire organ of Corti revealed that OHC *C1ql1* was expressed in a tonotopic gradient, but not in a commonly observed apex-to-base or base-to-apex pattern. The highest expression was in the 8–16 kHz region (Fig 2C and 2D). This region corresponds to the frequencies where mice have the greatest auditory sensitivity. However, even in this region, not all OHCs expressed *C1ql1*, and OHCs that expressed *C1ql1* had variable amounts of expression (Fig 2D). We noted no difference in the amount of mVenus signal in the different OHC rows (Fig 2D). mVenus signal was also visible in two additional unknown sources (Fig 2C, enlarged in panels 2F and 2G). The signal in panel F closer to the habenula perforata might come from cells in the osseous spiral lamina, the spiral limbus, or the inner sulcus. Whatever the source, it was present in a gradient, highest in the base and decreasing towards the apex. Panel G clearly reveals mVenus/*C1ql1*-positive soma near the cochlear modiolus. Neither of these unknown sources of mVenus signal overlapped with MAP2, suggesting they are not of any neuronal origin (not shown). Additional investigation is needed to identify the source of these signals. *C1ql1* appears to be the only member of the paralog family expressed in the cochlea; no expression was found for *C1ql2* or *C1ql4* by qRT-PCR, and no mVenus observed in the previously published *C1ql3*-mVenus reporter mouse [31].

*C1ql1* was found to be expressed in cochlear tissue harvested from adult mice [27, 28], but not from tissue harvested from neonates or developing mice [29, 30]. To investigate the timing of *C1ql1* expression, we examined *C1ql1* expression at earlier developmental time points. Surprisingly, we observed no *C1ql1* expression in the organs of Corti from 2-week-old mice (Fig 2H). Combined with above-mentioned gene expression profiling, we conclude that *C1ql1* is expressed in OHCs, but not in IHCs. Additionally, *C1ql1* is preferentially expressed in the region of greatest auditory sensitivity, and its expression is developmentally regulated. This developmental regulation is surprising; both C1QL1 and its close paralog C1QL3 have synaptogenic functions in the brain [31, 32, 37–39], but *C1ql1* expression is absent during the primary post-natal cochlear period of synaptogenesis [55]. This suggests that C1QL1 is not required for initial synaptogenesis in the cochlea.

### *C1ql1* is not expressed in the cochlear nucleus in the brain

**Fig 3:**
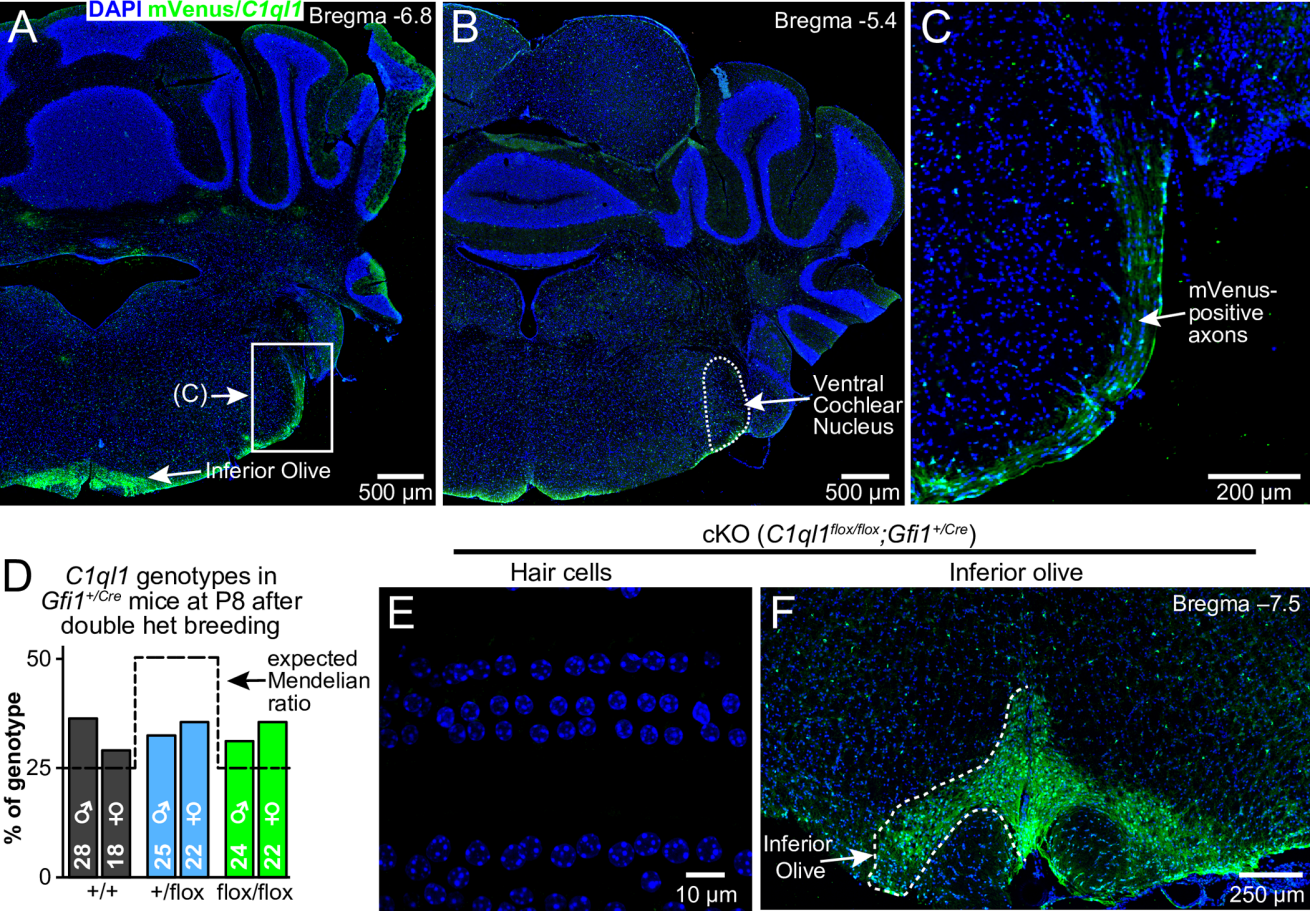
*C1ql1* is not expressed in cochlear nucleus and creation of *C1ql1* cKO mice. (A, B) Representative mVenus localization in coronal sections from adult *C1ql1*^*flox/flox*^ mouse hindbrain. (C) Magnified view of the boxed region in panel A. (D) Genotyping results from cross of double heterozygous parents. Only pups heterozygous for the *Gfi1*^*+/Cre*^ allele are graphed. Dashed line indicates the expected Mendelian ratio. (E) Representative image from a 10-week-old cKO (*C1ql1*^*flox/flox*^;*Gfi1*^*+/Cre*^) mouse taken at a similar frequency location as in panel 2E. (F) Representative mVenus localization in coronal section from adult cKO mouse hindbrain.

A prior analysis using *in situ* hybridization documented *C1ql1* expression in both the inferior olive and ventral cochlear nucleus of the hindbrain [33]. We examined adult brains from *C1ql1*^*flox/flox*^ mice, and similarly observed strong expression in the inferior olive (Fig 3A), but not in the cochlear nucleus (Fig 3B). An advantage of our mVenus knock-in strategy is that it distinguishes between the soma of a neuron and its axons. A close examination of the cochlear nucleus region revealed that mVenus-positive axons emanating from the neurons of the inferior olive travel adjacent to the ventral cochlear nucleus as they turn dorsally towards the cerebellum (Fig 3C). The *C1ql1*-expressing inferior olive neurons likely contain *C1ql1* mRNA in their axons and the previously observed *in situ* hybridization signal in this area was possibly mislabeled as *C1ql1* expression in the cells of the ventral cochlear nucleus [33].

### Development of *C1ql1* cKO mice

To study the function of *C1ql1* in OHCs, we used an intersectional genetic approach to create conditional knockout mice (cKO). The *C1ql1* allele described in Fig 2A was created in a similar manner as the previously published conditional *C1ql3* allele [31]. Cre recombinase excises an essential exon creating a null allele and silences expression of the mVenus reporter. In the cochlea, *Gfi1* is expressed in both hair cell types. We acquired mice harboring an allele (*Gfi1*^*Cre*^) to express Cre under the control of the *Gfi1* promoter (heterozygotes; *Gfi1*^*+/Cre*^) [48]. These Cre-driver mice were crossed to *C1ql1*^*flox/flox*^ mice to create double heterozygotes, which were interbred to create control (*C1ql1*^*+/+*^;*Gfi1*^*+/Cre*^) and cKO (*C1ql1* ^*flox/flox*^;*Gfi1*^*+/Cre*^) mice. A thorough analysis of *Gfi1-Cre* expression demonstrated that *Gfi1* is expressed in multiple locations outside of the auditory system, but only overlapped with *C1ql1* in the OHCs [48]. This suggests that any other cell type that expresses *C1ql1* will be unaffected in the cKO, and that Cre expression in cells that do not express *C1ql1* is irrelevant. *C1ql1* cKO mice were viable, fertile, and had no overt abnormal phenotype. cKO mice were not born in the normal Mendelian ratio as expected given the known reduced viability of the *Gfi1*^*Cre/Cre*^ mice. However, only considering the *Gfi1*^*+/Cre*^ genetic background, *C1ql1*^*flox/flox*^ mice were recovered at greater than the expected 25% percent of progeny, for unknown reasons (Fig 3D).

Examination of the OHCs from cKO adult cochleae showed no mVenus reporter expression in the OHCs (Fig 3E), consistent with our expectation that Cre-mediated recombination causes degradation of the entire transcript and a loss of mVenus translation. As the transcript is bicistronic, this strongly suggests that no C1QL1 protein can be made in the cKO OHCs. Examination of the hindbrain of cKO mice demonstrated that *C1ql1* expression is unaltered in the inferior olive neurons (Fig 3F). Also visible in the brain sections of Fig 3 are mVenus-positive cells outside the inferior olive and scattered in a roughly uniform manner throughout all brain regions. These cells were previously unappreciated and are of unknown identity, but possibly are parvalbumin-positive interneurons based on recent single-cell RNA sequencing [56]. Regardless, *C1ql1* expression in this cell type was also unaffected in the cKO genotype. We harvested total RNA from control and cKO cochleae and attempted to use qRT-PCR to demonstrate a lack of *C1ql1* transcript in cKO cochleae, but no such elimination was observed (not shown), likely because OHCs are not the only source of *C1ql1* expression in the cochlea (Fig 2C, 2F and 2G). The mVenus signal in the regions of Fig 2F and 2G persist in cKO cochleae (not shown), indicating these cells do not express *Gfi1*.

### Auditory physiological analysis of cKO mice

**Fig 4:**
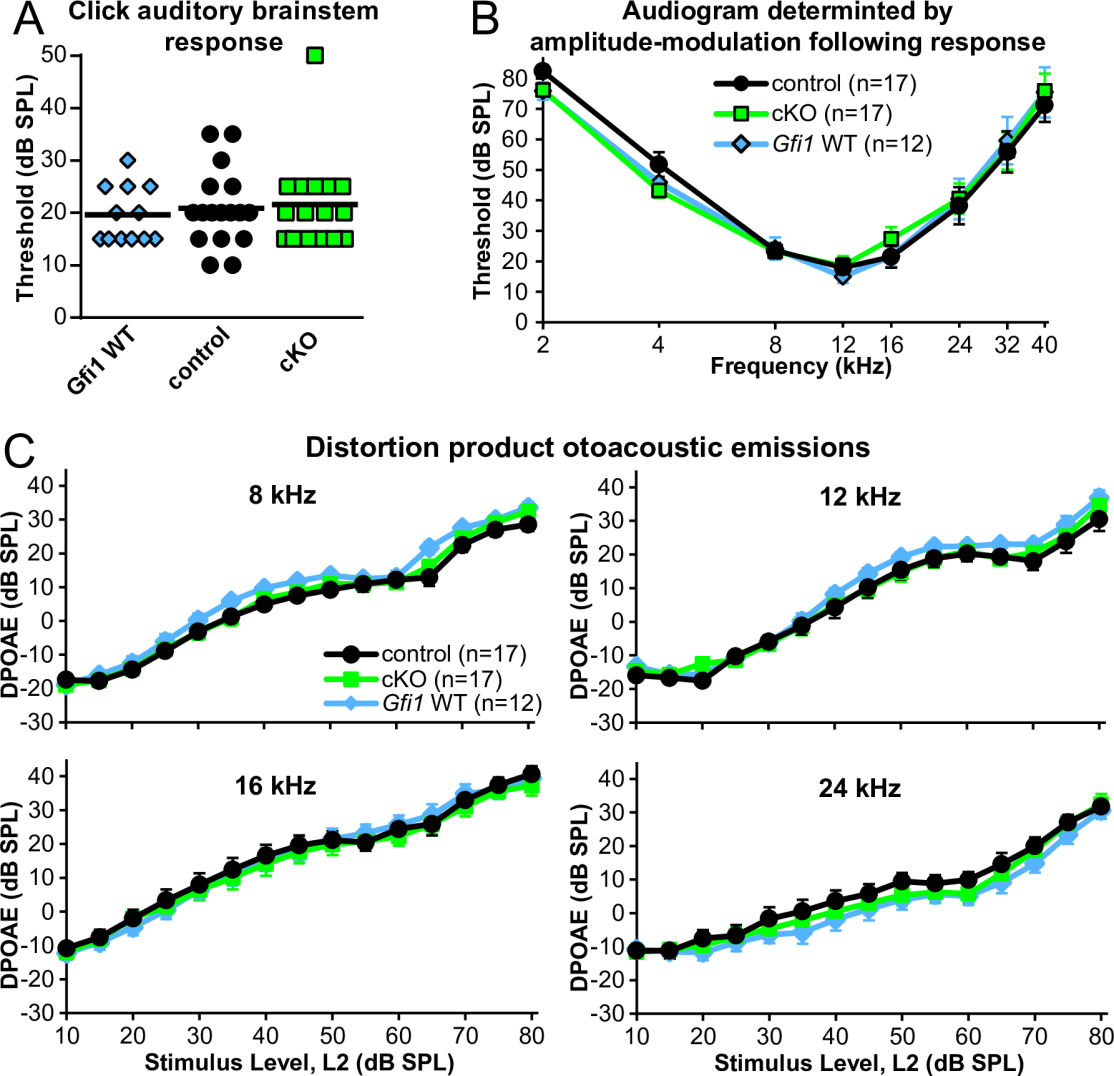
*C1ql1* cKO mice have normal auditory thresholds and OHC electromotility. (A) Click thresholds determined by ABR in the indicated genotypes. Each point is one mouse. Mice in this cohort were 7 weeks old. (B) Audiograms in the indicated genotypes produced using the AMFR method. Data are presented as mean ± SEM. Sample size indicated in graph legend. (C) DPOAEs recorded at the indicated center frequencies in the indicated genotypes. Data are presented as mean ± SEM. The center frequencies of 4 and 32 were also tested and revealed no differences between genotypes (not shown).

It is possible by an unknown mechanism that C1QL1 has a function to modulate OHC electromotility and thus influence auditory sensitivity. We tested if a lack of *C1ql1* expression changes the basic auditory physiology of a mouse. To test whether *C1ql1* cKO mice have altered auditory thresholds, we recorded auditory brainstem responses (ABR) in cKO and control genotypes in 7-week-old mice. The response to a broad spectrum of sound was tested using a click presented to mice at varying intensity levels, while electrodes placed under the scalp detected if the sound induced an auditory brainstem response. Mice heterozygous for the *Gfi1*^*Cre*^ allele were reported to have accelerated age-related hearing loss [57]; this is in addition to the well-known accelerated age-related hearing loss in the C57BL/6 strain [58]. Therefore, an additional control group of *Gfi1*^*+/+*^ mice was included. We found no threshold change between the tested genotypes (Fig 4A). To examine the auditory thresholds at specific frequencies of sound, audiograms were obtained by recording amplitude modulation following responses (AMFR). The AMFR is an electrophysiological response that is more sensitive and frequency-specific than the ABR, and is not reliant on any subjective evaluation [51, 52]. The recorded audiograms revealed no differences between genotypes (Fig 4B).

To test for deficits in OHC electromotility, we assayed distortion product otoacoustic emissions (DPOAEs). DPOAEs are created in the cochlea as distortions in the hair cell transduction process in response to two continuous tones (F_1_ and F_2_), which are reverse-transduced into mechanical distortions by OHCs, and back-propagated as pressure waves measurable in the ear canal with a microphone. There is a close relation between OHC function and DPOAE amplitude. We recorded DPOAEs induced by varying stimuli intensities at 2F_1_- F_2_, the strongest of the intermodulation distortion components. We did not observe any evidence for altered OHC electromotility in *C1ql1* cKO mice at any of the tested frequencies (Fig 4C). DPOAEs and auditory thresholds were similarly tested in a distinct cohort at 4 weeks old which yielded identical conclusions (not shown). Despite *C1ql1*’s expression pattern, these physiology results argue against the hypothesis that C1QL1 affects OHC electromotility and thus auditory thresholds.

### Acoustic trauma paradigm

**Fig 5:**
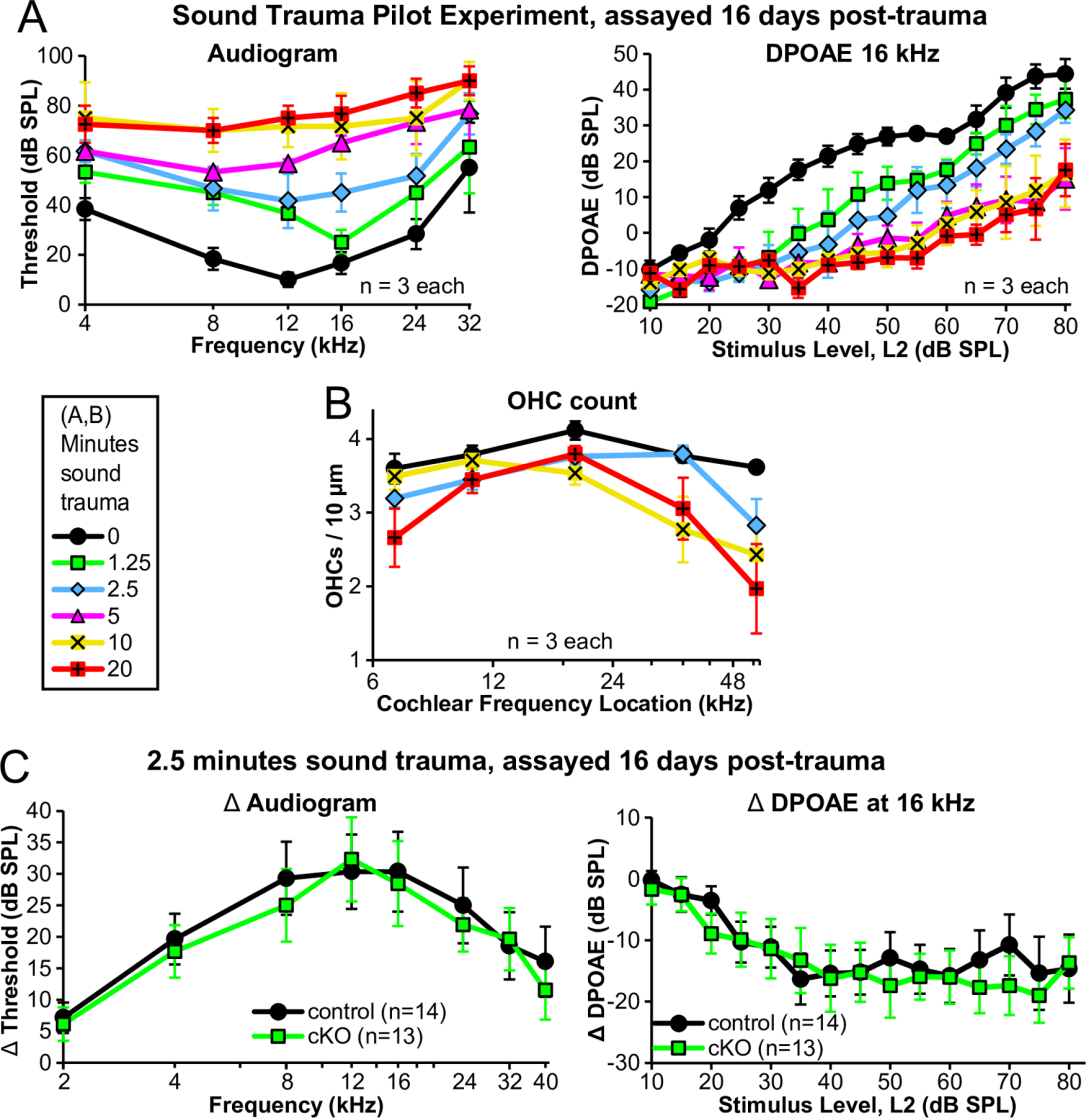
*C1ql1* cKO and control mice have indistinguishable susceptibility to acoustic trauma. (A, B) Auditory thresholds, DPOAEs, and OHC survival were tested on 8-week-old *C1ql1*^*flox/flox*^ mice after varying durations of acoustic trauma. 120 dB SPL of sound was presented at 8–16 kHz to anesthetized mice. N = 3 for each duration. Assays were conducted 16 days post-acoustic trauma. DPOAEs were also tested at 8, 12, and 24 kHz but these data did not add to the interpretation. (C) 2.5 min of acoustic trauma to 8-week-old control and cKO mice revealed no differences between genotypes in the audiogram or DPOAEs 16 days post-trauma. DPOAEs were also tested at 8, 12, and 24 kHz, with no differences between genotypes observed (not shown). Data are presented as mean ± SEM.

We next asked if C1QL1 is involved in the protection against OHC death. Before we could test the *C1ql1* cKO mice for deficits in OHC protection during acoustic trauma, we conducted a pilot experiment to establish an acoustic trauma paradigm and to determine the ideal acoustic stimulus duration in our laboratory. We exposed anesthetized 8-week-old mice to 120 dB sound pressure level (SPL) of octave-band noise, 8–16 kHz, for varying durations of time between 0 and 60 minutes. 16 days later, mice were evaluated for the extent of permanent auditory threshold increase and loss of OHC function. Following physiology assays, cochleae were dissected and the extent of OHC death was quantified at representative frequency locations. We found that after 10–20 minutes of this acoustic trauma, a ceiling of threshold increase was reached, suggesting that all OHCs were either dead or non-functional at this point (Fig 5A; durations greater than 20 minutes omitted for clarity). OHC quantification from dissected cochleae revealed surprisingly intact OHC numbers, suggesting that the acoustic trauma mainly rendered OHCs non-functional, as opposed to ablating them (Fig 5B).

Based on this pilot experiment, we chose 2.5 minutes of acoustic trauma as an approximate ‘halfway’ duration. The same cohort of mice physiologically tested in Fig 4 was subjected to this trauma at 8 weeks old and similarly assayed again 16 days later. The increase in thresholds and decrease in DPOAE magnitudes are plotted in Fig 5C. *C1ql1* cKO mice were not found to have a difference in their susceptibility to this acoustic trauma. It remains possible that testing other durations of acoustic trauma, other ages of mice, or a different type of sound exposure might reveal differences in the cKO mice.

### C1QL1 may promote OHC afferent synapse completion

**Fig 6:**
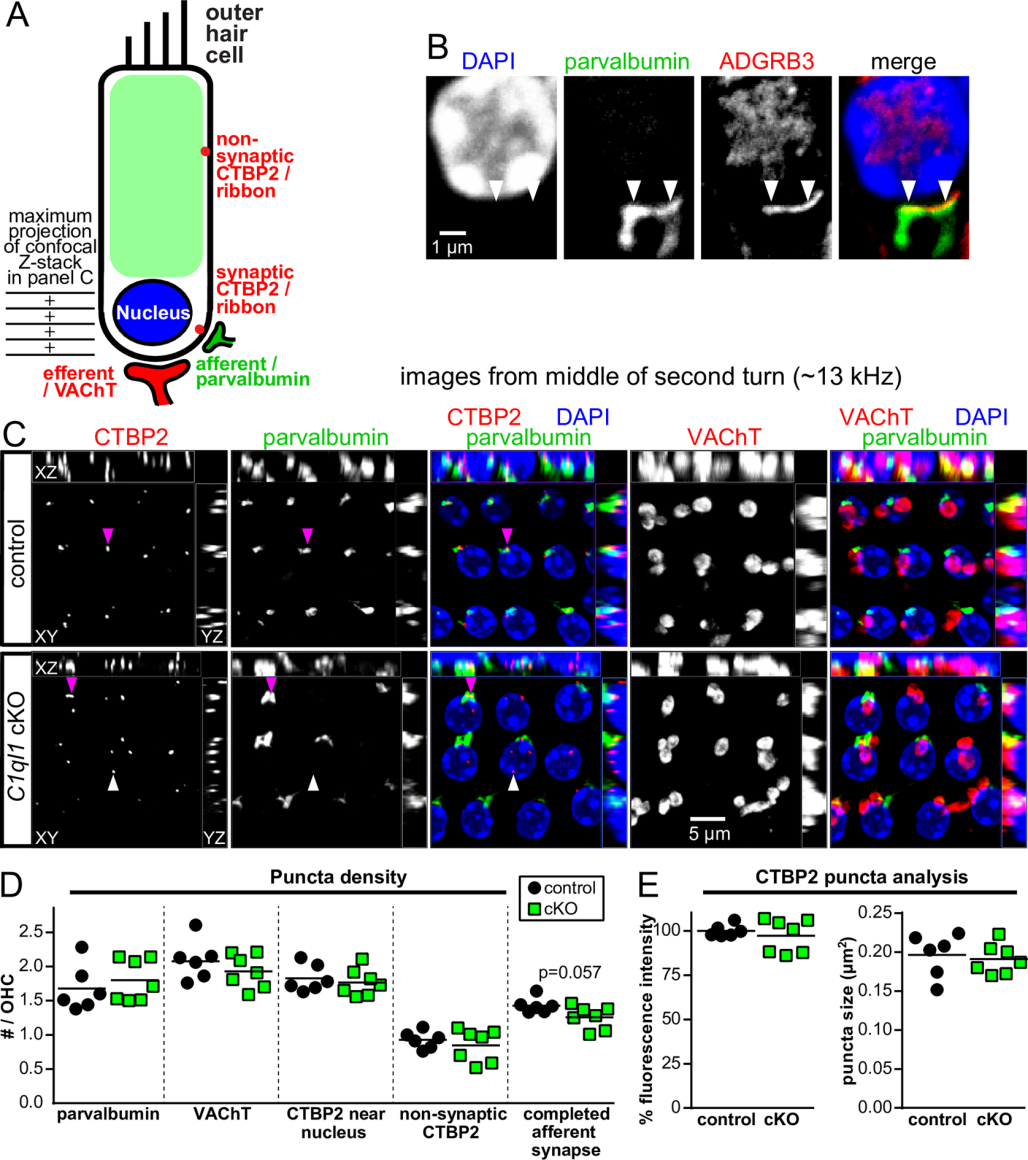
*C1ql1* cKO OHCs have normal synaptic marker density, but may have deficits in afferent synapse completion. (A) Drawing demonstrating the locations of the parvalbumin-positive afferent post-synapses, the VAChT-positive efferent synapses, and the 2 types of CTBP2-positive pre-synaptic ribbons. At left is the region of the OHCs depicted in the representative pictures in panel C. (B) Representative immunofluorescence of an OHC at a single confocal plane for post-synaptic proteins parvalbumin and ADGRB3. Arrowheads point to each of the apparent afferent synapses. Note that the antibody for ADGRB3 was previously published to work for western blots [49] and we do not have an *Adgrb3* KO control to verify the specificity of the signal; thus this staining should be interpreted with caution. The ADGRB3 signal in the nucleus is likely non-specific. (C) Representative confocal z-stack maximum projection from indicated genotypes at ~13 kHz region of organ of Corti. The maximum projection in each orthogonal plane included as indicated. Purple arrowheads indicate a CTBP2 puncta with an adjacent parvalbumin puncta; white arrowheads indicate a CTBP2 puncta with no apparent associated post-synaptic side (‘orphan ribbon’). Note only basal regions of OHCs are depicted here, therefore, non-synaptic CTBP2 puncta and the cytoplasmic parvalbumin signal in OHCs are not visible. Mice were 10–11 weeks old. See also the S1 Movie. (D) Quantification of the indicated synaptic puncta density per OHC. Z-stacks of entire OHCs were examined plane-by-plane, not just the region depicted in panel C. A completed afferent synapse was defined as a CTBP2 puncta with a juxtaposed parvalbumin puncta. Each data point shown is the mean of 1 mouse. Statistical test: Students t-test. (E) CTBP2 puncta from maximum projections of the entire OHC were quantified for both surface area and relative fluorescence intensity.

The OHC afferent synapse is a true glutamatergic synapse, stimulated by OHC depolarization, with post-synaptic AMPA receptors to initiate an action potential in the type II SGN [59, 60]. In the CNS, C1QL1 is a protein secreted into the synaptic cleft [37]. Thus, it is reasonable to predict that C1QL1 protein will be secreted into the synaptic cleft of the OHC afferent synapse. This leads to the prediction that knocking out *C1ql1* from OHCs will cause a selective loss of OHC afferent synapses. To determine if loss of C1QL1 causes a change in the density of OHC afferent or efferent synapses, we detected synapses by immunofluorescence on dissected organs of Corti using established markers: CTBP2 to mark the pre-synaptic side of afferent synapses, parvalbumin to mark the post-synaptic side of afferent synapses, and vesicular acetylcholine transporter (VAChT a.k.a. VAT) to mark the pre-synaptic side of efferent synapses (Fig 6A).

Parvalbumin is a relatively new marker to use in this context. Distinct from the CNS, parvalbumin protein is present in the cytoplasm of both hair cell types, and fills the neurites of both types of SGN [10, 61, 62]. Parvalbumin signal overlapped with the expected post-synaptic localization of ADGRB3 (Fig 6B) and was juxtaposed to pre-synaptic CTBP2 (Fig 6C). See also the S1 Movie. We also observed that the cytoplasmic signal of parvalbumin in OHCs was present in a tonotopic gradient, highest in the apex, faintly in the middle, and lowest in the base (not shown). In the representative pictures shown in Fig 6C, Z-stacks were created to omit the faint parvalbumin signal in the OHC cytoplasm.

In our histological analysis, mice were not previously exposed to acoustic trauma. As previously observed [10, 63], there not only exist CTBP2 puncta at the basal pole of the OHC adjacent to its corresponding post-synapse, but also higher up on the soma, with no post-synapse (Fig 6A). The function of these “non-synaptic” pre-synaptic terminals is unknown, and they were counted separately. Quantification of each of the synaptic markers per OHC at the ~13 kHz region showed no changes in the cKO genotype (Fig 6D). We additionally quantified the size and fluorescence intensity of the CTBP2 puncta but observed no differences in the cKO genotype (Fig 6E).

If C1QL1 has a function in synaptic adhesion, then loss of C1QL1 might reduce the likelihood of juxtaposed pre- and post-synaptic membranes. To examine this possibility, we quantified the number of ‘completed synapses,’ defined as the number of CTBP2 puncta with a juxtaposed parvalbumin puncta (purple arrowheads in Fig 6C). Interestingly, a subset of the cKO mice showed a clear reduction in the density of completed afferent synapses, suggesting that C1QL1 protein has a function in afferent synapse maintenance (Fig 6C; white arrowheads point to a CTBP2 puncta with no juxtaposed post-synapse). However, combining results from all tested organs of Corti did not yield a statistically significant reduction (Fig 6D), and this conclusion must be interpreted with caution.

### Auditory-dependent behavioral analysis

**Fig 7:**
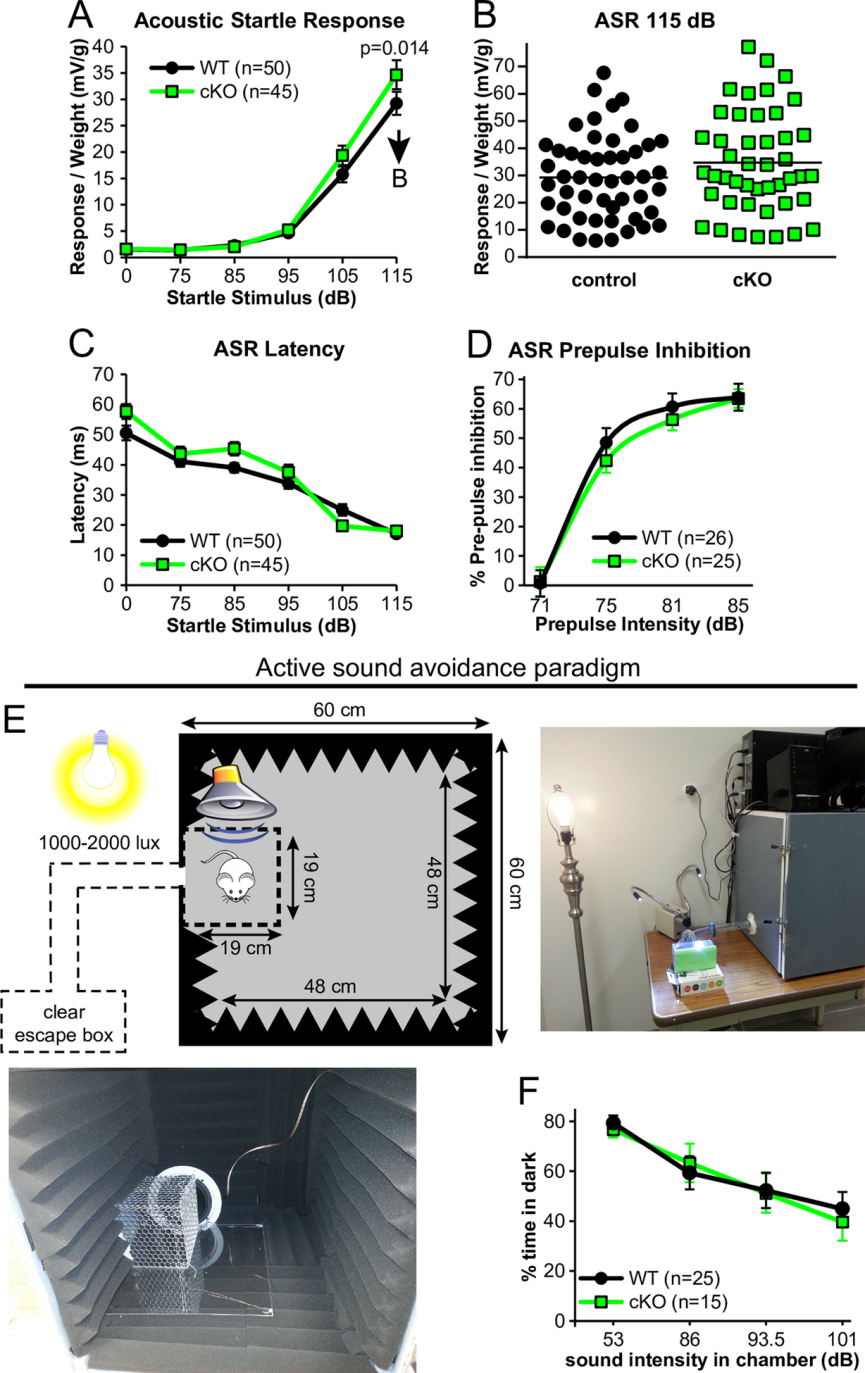
Auditory-dependent behavioral analysis of *C1ql1* cKO mice. (A) ASR at the indicated decibel levels. Mice were 7–10 weeks old. Statistical test: repeated measures 2-way ANOVA with Bonferroni’s post-hoc test. (B) The data presented for the 115 dB SPL sound level in panel A is reproduced here, but displaying each data point. Each point is 1 mouse. (C) Latency of ASR at the indicated decibel levels. (D) Pre-pulse inhibition of the ASR (consistent 115 dB SPL startle) at the indicated pre-pulse decibel levels. (E) Construction of a custom-built behavior apparatus to test active sound avoidance in mice. (F) Results from the indicated genotypes in sound avoidance behavior at varying sound intensity levels. Mice were 10–11 weeks old.

Since *C1ql1* cKO mice did not show altered auditory threshold sensitivity, we tested their behavioral responses to higher intensity, supra-threshold sounds. We tested the acoustic startle response (ASR) which is a measurable, unconscious ‘flinch’ reflex due to a sudden and unexpected sensory stimulus. We found that with stimuli of 115 dB SPL (50 ms broadband), *C1ql1* cKO mice had an enhanced ASR compared to controls (Fig 7A). Although a statistically significant result was obtained, the ASR typically shows high variability and there may be other factors that could add to the observed differences in the startle response of these groups (individual points for each mouse plotted in Fig 7B). The latency of the startle response was also measured, but revealed no statistically significant differences (Fig 7C). We also measured pre-pulse inhibition to the ASR, showing no statistically significant differences (Fig 7D), indicating normal sensorimotor gating.

In an attempt to more directly assay cKO mice for behavioral changes suggestive of altered responses to perceived loud sounds, we built a custom apparatus to assay active sound avoidance behavior in mice, inspired by a rat sound avoidance apparatus in [53] (Fig 7E). A loudspeaker was placed inside a dark, sound-attenuated chamber facing a smaller inner cage where a mouse was placed. The speaker was programmed to emit 8–16 kHz sound at mean intensity levels of 86, 93.5, or 101 dB SPL as measured from inside the inner cage. The mouse was allowed the opportunity to leave the inner chamber through a hole connected to a transparent ‘escape’ box. Bright lights illuminated the escape box. When the 101 dB SPL sound was emitted from the speaker, the sound intensity level in the escape box was 76 dB SPL for a ~316 fold decrease in sound intensity.

When the speaker was off, mice chose to spend the majority (~80%) of their time inside the dark box, due to a mouse’s innate aversion to bright lights (Fig 7F). The protocol for habituation and sound stimulus testing was similar to [53]. In brief, after 3 habituation days with the speaker off, the speaker is turned on to emit sound at various intensity levels presented over 3 days in a random order. The underlying assumption is that if a mouse finds the sound intensity level uncomfortable or unbearable, the mouse will re-locate outside the dark box. As expected, control mice chose to spend increasingly more time outside the dark box as the sound intensity level was increased. However, the behavior of cKO mice was indistinguishable from the controls in this assay.

## Discussion

In this manuscript, we present the creation and first uses of a novel *C1ql1* allele that combines both conditional knockout potential and a fluorescent reporter to detect *C1ql1* gene expression. This reporter allele allowed us to make several key observations. First, we resolved a discrepancy in previous expression profiling studies regarding the expression of this gene in hair cells: the gene is not expressed in neonatal OHCs, but is expressed in adult OHCs. Second, this reporter allele revealed an unexpected and extremely interesting expression pattern in which *C1ql1* is expressed in OHCs and never in IHCs, and the extent of expression correlates with cochlear regions with the greatest auditory sensitivity. Third, we found that *C1ql1* is expressed in at least 1 additional cell type in the cochlea of unknown identity. Fourth, we show that *C1ql1* is not expressed in the cochlear nucleus of the brain as was previously reported [33]. Fifth and lastly, we found *C1ql1* is expressed in a previously unappreciated cell type scattered roughly uniformly throughout the brain that is of unknown identity, but possibly are parvalbumin-positive interneurons [56].

To study the function of C1QL1 in OHCs, we created and characterized novel *C1ql1* OHC-specific cKO mice. The *C1ql1* OHC expression was primarily in the parts of the cochlea that represent the frequencies with the lowest auditory thresholds in the mouse, however, it does not appear that C1QL1 affects auditory sensitivity or OHC electromotility. A lack of influence on auditory sensitivity would not be inconsistent with our hypothesis regarding C1QL1 secretion and localization. Given the previous literature on the C1QL protein family, we hypothesized that C1QL1 protein would be secreted pre-synaptically from the OHCs and reside in the afferent synaptic cleft. Furthermore, we predicted that loss of C1QL1 would cause a deficit in the formation or maintenance of the OHC to type II SGN synapse. If true, then an OHC-specific KO of *C1ql1* would allow us to selectively probe the function of this mysterious synapse and the type II afferents that they activate.

The prevalent hypothesis about the function of the OHC afferent synapse and type II SGN is that they are useful in the sensation of high-level, potentially tissue-damaging sounds. Intense noise exposure is one of the most frequent causes of sensorineural hearing loss, and OHCs are the most vulnerable to damage [64, 65]. It is thought that the type II SGNs may function as auditory nociceptors [16, 25] since they resemble the small, unmyelinated C-fibers of the dorsal root ganglion that carry nociceptive information to the spinal cord [3]. A number of studies suggest that high-level acoustic inputs are required to initiate an action potential in the type II SGNs [24, 25, 60, 66–68]. Moreover, ATP that is released from damaged cells in the organ of Corti can directly excite type II afferents [26], suggesting that the type II afferents are capable of reporting tissue damage in the organ of Corti to the brain. Whether OHC afferent stimulation is sufficient to induce a conscious perception of pain via pain pathways is still unclear.

Recently, evidence has emerged that the type II SGN also may be sensitive to non-damaging sounds [69, 70]. It is unclear what role, if any, they may play in normal hearing. However, with a wide dynamic range, the type II SGNs may better contribute to an early warning circuitry to prevent cochlear damage. It is likely that central circuitry connects the type II afferents to the OHC efferents resulting in protection of OHCs from damage. Negative feedback from the medial olivocochlear efferent reflex provides some protection against OHC damage from acoustic trauma [71–76]. Thus, the activity of the type II SGNs before the sound is damaging may help protect the cochlea through the initiation of the medial olivocochlear reflex and other signals transmitted to the brain.

Our unexpected finding that *C1ql1* was not expressed at a peak period of synaptogenesis in the cochlea argues strongly against a role for C1QL1 in synaptogenesis, although it still might have a function in synapse maintenance. This maintenance could be accomplished by C1QL1 participating in a trans-synaptic adhesion complex similar to that predicted for C1QL3 in the CNS or by engaging in some post-synaptic signaling event [34]. We show here that the GPCR ADGRB3, which binds all C1QL paralogs, is located post-synaptically at the afferent synapses, making this a possibility. However, our histological investigation into OHC afferent synapse maintenance yielded inconclusive results: no change in the number of pre-synaptic ribbons or post-synaptic markers, but a trend for a reduction of ‘completed’ afferent synapses defined as adjacent pre- and post-synaptic markers. An additional possibility is that C1QL1 alters the physiology of this synapse, without changing synapse density. C1QL2 and C1QL3 bind to an ionotropic glutamate receptor (kainate receptor GluK2) [40], and this protein is present at the OHC afferent synapse [77]. Given the structural similarity between C1QL1 and C1QL3 [35], C1QL1 likely binds to GluK2 as well, and this interaction may modulate the synapse’s physiology.

A limitation of this study was the inability to immuno-localize the C1QL1 protein. Unfortunately, we were unable to acquire an antibody capable of immuno-localization in the cochlea, thus the predicted C1QL1 localization to the OHC afferent synapse remains conjecture. An alternative localization is secretion into the efferent synaptic cleft; however, no evidence was obtained for altered function of the medial olivocochlear efferents. Alternatively, C1QL1 might be secreted to signal to a distantly located cell type, with no synaptic functions.

Regardless of the true C1QL1 localization after OHC secretion, no compelling phenotypes were found in the cKO mice using standard auditory physiology assays or supra-threshold behavioral assays. We potentially observed an enhanced ASR in cKO mice compared to control genotypes; this could be critical evidence as multiple studies suggest that hyperacusis causes an increase in the ASR assay [78, 79]. However, we note the large variability in our ASR results, warranting caution. Moreover, ASR has limitations, *e*.*g*., enhanced ASR is also reported in tinnitus [79]. Our additional assay to measure auditory nociception by measuring changes in behavior when exposed to intense sounds did not detect a phenotype.

In summary, we report an intriguing expression pattern for the *C1ql1* gene in OHCs in the adult cochlea using a novel *C1ql1* reporter allele, potentially prompting creation of new tools to study the function of OHCs and type II SGNs. We created and characterized novel *C1ql1* OHC-specific cKO mice, but our numerous assays did not reveal any compelling phenotypes, and the function of C1QL1 in the cochlea remains unknown. Given the unexpected late onset of *C1ql1* expression, it would be of interest to examine histology, auditory sensitivity, and behavior in aged cKO mice. Unfortunately, the early-onset hearing loss in our choice of mouse strain and Cre-driver allele prevented such an analysis in the present study. Using an alternative strain and Cre-driver allele is an attractive opportunity for future investigation.

## Supporting information

S1 Movie Movie of confocal z-stack of hair cells. Representative confocal Z-stack of both hair cell types. Anti-CTBP2 is red, anti-parvalbumin is green, anti-VAChT is purple, and nucleus stained blue with DAPI.
(WMV)
