# Correction: *C1ql1* is expressed in adult outer hair cells of the cochlea in a tonotopic gradient

**DOI:** 10.1371/journal.pone.0333506

**Published:** 2025-09-29

**Authors:** Joyshree Biswas, Robert S. Pijewski, Rohit Makol, Tania G. Miramontes, Brianna L. Thompson, Lyndsay C. Kresic, Alice L. Burghard, Douglas L. Oliver, David C. Martinelli

There is an error in panel B of Fig 3. The arrow is pointing to the incorrect brain region. Please see the correct Fig 3 here.

**Fig 3:**
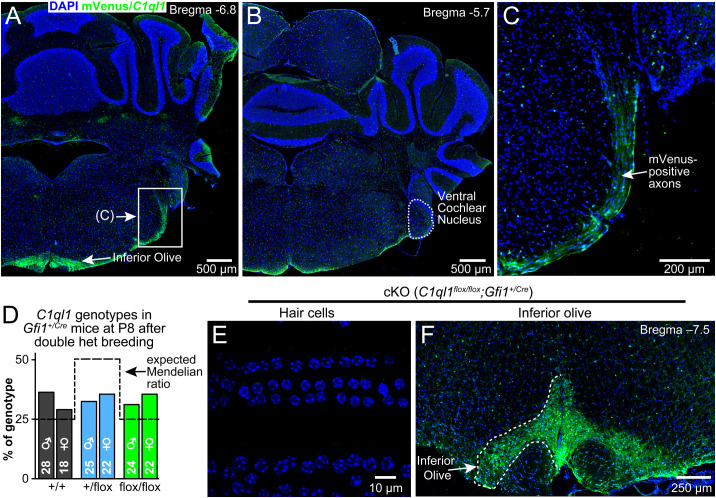
*C1ql1* is not expressed in cochlear nucleus and creation of C1ql1 cKO mice. **(A, B)** Representative mVenus localization in coronal sections from adult *C1ql1*^*flox/flox*^ mouse hindbrain. **(C)** Magnified view of the boxed region in panel **A. (D)** Genotyping results from cross of double heterozygous parents. Only pups heterozygous for the *Gfi1*^+*/Cre*^ allele are graphed. Dashed line indicates the expected Mendelian ratio. **(E)** Representative image from a 10-week-old cKO (*C1ql1*^*flox/flox*^*;Gfi1*^+*/Cre*^) mouse taken at a similar frequency location as in panel 2E. **(F)** Representative mVenus localization in coronal section from adult cKO mouse hindbrain.
